# Assessing the risk for falls among Portuguese community-dwelling stroke survivors. Are we using the better tools? Observational study

**DOI:** 10.1097/j.pbj.0000000000000160

**Published:** 2022-06-17

**Authors:** Carla Pimenta, Anabela Correia, Marta Alves, Daniel Virella

**Affiliations:** a Physiotherapy, Hospital Curry Cabral, Centro Hospitalar Universitário Lisboa Central and Teaching and Research Unit of Physiotherapy and Rehabilitation, Escola Superior de Tecnologia da Saude de Lisboa, Instituto Politécnico de Lisboa, Portugal; b Epidemiology and Statistics Office of the Research Unit, Centro Hospitalar Universitário Lisboa Central, Portugal

**Keywords:** community-dwelling, cross-sectional study, falls, postural balance, risk factors, stroke

## Abstract

This study assesses the estimation of the risk for falls among community-dwelling stroke survivors referred for ambulatory physiotherapy and explores factors that affect the risk.

**Methods:**

Observational, cross-sectional with nested case-control study, of individuals, referred to physiotherapy less than 12 months after stroke and able to walk independently. Berg Balance Scale, Timed Up and Go Test, and the Motor Assessment Scale were applied. Berg Balance Scale ≤45 or Timed Up and Go Test > 14 were used to estimate the risk for falls. The discrimination ability of the estimation was assessed. Alternative models were explored by logistic regression analysis.

**Results:**

One hundred sixty-seven patients fulfilled the inclusion criteria. Patients were 21 to 87years old (median 66), 98 men (58.7%), and in 133 (79.6%) the stroke occurred in the last 6 months. Falls were reported by 78 (46.7%) of the patients but 139 (83.2% [95%CI 76.84–88.14]) were estimated as having risk for falls. The discrimination ability of the estimation of the actual occurrence of falls by Berg Balance Scale ≤45 or Timed Up and Go Test >14 was 55% (95%CI 47.5–62.4). The actual occurrence of falls was associated only with Motor Assessment Scale, as a protective factor. The discrimination ability of the estimation of the actual occurrence of falls by Motor Assessment Scale alone was area under the curve 0.69 (95%CI 0.60–0.77).

**Conclusions:**

Different tools with better performance are needed to identify the risk for falls after stroke.

## Introduction

Stroke is the most common neurological condition in adulthood that leads to motor disability;^1^ those who survive a stroke many never fully recover and the disabilities can range from mild to severe.^2^ With the aging of the population, the incidence of stroke has increased worldwide,^3^ although stroke mortality rates have been reduced with the advancements in acute healthcare.^2^

Compromised mobility is one of the reasons for admission for inpatient rehabilitation after stroke^4^; most of the patients with moderate or severe impairments, in the sub-acute phase of stroke, typically attend rehabilitation as an in-patient. Balance and gait disorders are common; they have a significant impact on functional autonomy, overall recovery, and the quality of life of stroke survivors,^4,5^ thus making the restoration of ambulation a significant part of the functional recovery following stroke.^4^

Lamb et al associated balance and mobility problems with the occurrence of falls among stroke survivors in the community.^6^ Falls are common among stroke survivors in all stages of stroke, occurring in the acute, rehabilitative, or chronic phases,^7^ although 1 study reports that most falls occur within 2 months after discharge from rehabilitation.^8^

Consequences of falls include minor or serious injuries or even death, reduced mobility, functional limitations, decreased activity, and fear of falling.^7^ As patients with poor ambulation capability and reduced balance are more likely to have a high concern with falling, interventions that improve functional ambulation and balance have a critical role in preventing falls.^3^

One in 5 post-stroke patients experienced a fall after discharge from an inpatient rehabilitation unit.^9^ During rehabilitation, the physiotherapy assessment should identify the risk of falling, to select strategies that allow minimizing this risk.^9^ Homecoming is useful for the rehabilitation of the patient by the reintegration in the family and the social and environmental network but leads to an increased risk for falls because the patient is no longer in a restricted, protected environment.

The primary objective of this study was to assess the performance of the estimation of the risk for falls among community-dwelling stroke survivors, using a simple tool based on the usual assessments of balance. The secondary objective was to explore factors associated with the risk for falls.

## Methods

This observational, cross-sectional study with a nested case-control study was performed in the physiotherapy department of a tertiary care hospital in Lisbon (Hospital Curry Cabrai - Centro Hospitalar Universitário Lisboa Central). A referral period of 5 years was considered (between July 1, 2014 and June 31, 2019). This study was authorized by the Clinical Director of the Centro Hospitalar Universitário Lisboa Central, with the approval of the Ethics Committee (Proc 140/2012), for not presenting ethical objections, complying with the standards of good clinical practice, and respecting the World Medical Association Declaration of Helsinki. To report this study was considered the guidance of the STrengthening the Reporting of OBservational studies in Epidemiology (STROBE statement).

The participants were community-dwelling adult patients with an autonomous walk, referred to the physiotherapy department outpatient clinic after a stroke diagnosed in the previous 12 months. Eligible patients were able to walk independently for 3 m; they might use any walking assistive device but no help from another person (score 3 of the walking assessment item in Motor Assessment Scale).^10^ All participants provided written informed consent.

Demographic and clinical data were collected, including age, gender, and date, anatomic location, and etiology of the stroke. Participants were inquired about the occurrence of episodes of falls after stroke. Three functional tools to assess function, walking, and balance were applied: Berg Balance Scale, Timed Up and Go Test, and the Motor Assessment Scale.

Berg Balance Scale is a functional tool to evaluate the dynamic balance, consisting of 14 tasks^11^ that correspond to activities of daily living.^12^ The tasks requested are of increasing difficulty with progressive reduction of the support base; 3 dimensions are evaluated: position maintenance, postural adjustment during voluntary movement, and reaction to external disturbances.^12^ The score ranges from 0 to 56 points, where 56 represents the best performance.

Timed Up and Go Test quantifies the functional mobility in seconds, recording the time spent performing the task of getting up from a standard chair with armrests (approximately 46 cm in height), walking a linear path at a comfortable and safe pace until a line on the ground 3 m away, change direction, walk the opposite way, return to sit, and resting the back on the same chair. The usual footwear should be worn; the use of walking assistive devices must be recorded. Shorter time indicates better functional performance. Timed Up and Go Test is considered a very useful instrument to evaluate the functional mobility in individuals with stroke with autonomic gait,^13–15^ it allows to evaluate the agility since it involves not only the capacity for walking but also the change of direction and the tasks of standing and sitting.^16,17^ Timed Up and Go Test allows the identification of the risk for fall in the elderly^18,19^ and in individuals with stroke.^14,19^

Motor Assessment Scale is a functionality scale for stroke patients.^10^ It is based on the evaluation of the performance of functional tasks instead of focusing only on isolated patterns of movement.^20^ It comprises 8 activities that are scored on 7 levels (from 0 to 6), from worst to best performance and claim to be hierarchical. This organization, in addition to making the scale appealing, reduces the time of application. Motor Assessment Scale is a useful tool for the physiotherapist since the score directly reflects the objectives of his intervention,^21^ with known validity, reliability, and ease of application.^22,23^ Therefore, it is widely used as an instrument for evaluating functionality, as a measure of the results of the intervention,^24^ as an aid in functional prognosis, or as an inclusion criterion in clinical trials.^25–29^

To minimize observer bias, in the first week of treatment, the clinical data were collected and the instruments were systematically applied following a standardized protocol by 2 experienced physiotherapists.

Patients estimated as having risk for falls were considered as cases; the remainders were the controls. The estimated risk for falls was defined as Berg Balance Scale ≤45^11^ or Timed Up and Go Test >14.^15,30^ The prevalence of risk for falls was estimated with 95% confidence intervals (CI).

To explore factors associated with the estimated risk for falls and to the actual occurrence of falls, multivariable logistic regression modeling was performed after identifying by univariable analysis the significantly associated variables eligible for inclusion; adjusted odds ratio (OR) was estimated with 95%CI.

The ability of the estimation of the risk for falls to identify the actual occurrence of falls was assessed by positive and negative predictive values and overall discrimination rate. The discrimination ability of the obtained model for the actual occurrence of falls was assessed by the area under the curve (AUC) with 95%CI.

Statistical analysis was performed using Epinfo (Dean AG, Sullivan KM, Soe MM. OpenEpi: Open Source Epidemiologic Statistics for Public Health, www.OpenEpi.com) and SPSS® 22.0 (SPSS® for Windows, Rel. 22.0.1. 2013. SPSS® Inc.,Chicago,IL, USA).

## Results

**Figure 1:**
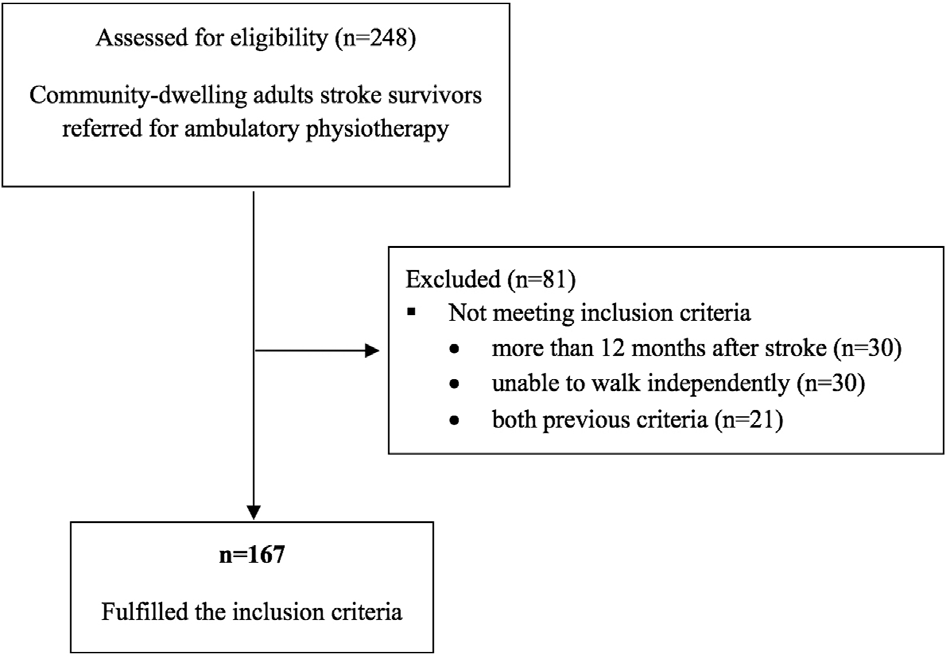
Flow diagram for sample selection.

During the referral period, 248 stroke survivors were sent for ambulatory treatment in the physiotherapy department (Fig. 1); 81 did not fulfill the inclusion criteria (the stroke had occurred more than 12 months earlier and/or the patient did not walk independently).

The 167 participants were 21 to 87 years old (median 66), 98 (59%) were men, the stroke occurred in the last 6 months in 133 (80%), it was ischemic stroke in 137 (82%) and it was not a first stroke event in 36 (12%).

Falls were reported in 78 (46.7% [95%CI 39.30–54.26]) of the patients. Estimated risk for falls (as defined above) was identified in 139 patients (83.2% [95%CI 76.84–88.14]); 111 cases were identified by both criteria simultaneously (Berg Balance Scale and Timed Up and Go Test), 15 only by Berg Balance Scale and 13 by Timed Up and Go Test alone.

The description of the functional assessment of the patients by Berg Balance Scale, Timed Up and Go Test, and the Motor Assessment Scale is presented in Table 1.

**Table 1:** Functional assessment of the patients by Berg Balance Scale, Timed Up and Go Test, and Motor Assessment Scale, discriminated by the estimated risk for falls

	Total sample n = 167	Cases (estimated risk for falls) n = 139	Controls (no estimated risk for falls) n = 28
BBS median (min.-max.) points	39 (7–55)	37 (7–51)	50 (46–55)
TUG median (min.-max.) seconds	18 (7–88)	19 (11–88)	11 (7–14)
MAS median (min.-max.) points	35 (11–48)	34 (11–46)	43 (32–48)

BBS, Berg Balance Scale; MAS, Motor Assessment Scale; TUG, Timed Up and Go Test.

Patients identified as having risk for falls were more likely to be female and older. The estimated risk of falls was positively associated with the time lag after stroke and negatively associated with the walking ability (Motor Assessment Scale) (Table 2). The multivariable logistic regression model for the estimated risk of falls identified gender (female adjusted OR 6.3 [95%CI 1.3–30.1]; *P* = 020) and age (for each increase of 1year adjusted OR 1.1 [95%CI 1.0–1.1]; *P* = .002) as risk factors and Motor Assessment Scale as a protectivefactor(for each increase of 1 unit adjusted OR 0.8 [95%CI 0.7–0.9]; *P* <.001).

**Table 2:** Demographic, clinical, and functional characteristics of the patients and their bivariable association with being identified as an estimated risk for falls (crude odds ratio).

	Total sample n = 167	Cases (estimated risk for falls) n = 139	Controls (no estimated risk for falls) n = 28	OR (95%CI) *P* value
Age (Years); n (%)				
Younger than 65 years old	77 (46.1%)	56 (40.3%)	21 (75.0%)	
65 years old and older	90 (53.9%)	83 (59.7%)	7 (25.0%)	4.4 (1.8–11.2) *P* = .001
Gender; n (%)				
Female	69 (41.3%)	64 (46.0%)	5 (17.9%)	3.9 (1.4–10.9) P = .009
Male	98 (58.7%)	75 (54.0%)	23 (82.1%)	
Time lag after stroke (months); n (%)				
< 3 months	55 (32.9%)	41 (29.5%)	14 (50.0%)	reference
3–6 months	78 (46.7%)	68 (48.9%)	10 (35.7%)	2.3 (0.9–5.7) *P* = .066
6–9 months	20 (12.0%)	16 (11.5%)	4 (14.3%)	^*^2.6 (0.8–8.6) *P* = .127
9–12 months	14 (8.4%)	14 (10.1%)	-	
Walking ability^#^ (by the walking assessment item of MAS); n (%)				
3	70 (41.9%)	69 (49.6%)	1 (3.6%)	168.7 (20.0–1406.6) *P*< .001
4	41 (24.6%)	38 (27.3%)	3 (10.7%)	31.0 (7.6–126.6) *P* < .001
5	25 (15.0%)	23 (16.5%)	2 (7.1%)	28.1 (5.5–144.9) *P* < .001
6	31 (18.6%)	9 (6.5%)	22 (78.6%)	reference
Patients with reported episodes of fall; n (%)				
Yes	78 (46.7%)	71 (51.1%)	7 (25.0%)	3.1 (1.3–7.8) *P* = .015
No	89 (53.3%)	68 (48.9%)	21 (75.0%)	

CI, confidence interval; MAS, Motor Assessment Scale; n, number; OR, odds ratio.

* Collapsed time lag class 6–9 months.

# Walking ability: 3. Walks 3 m without assistance but with an assistive device; 4. Walks 5m without a device or assistance in 15 seconds; 5. Walks 10 m without assistance or a device. Is able to pick up a small object from the floor with either hand and walk back in 25 seconds; 6. Walks up and down 4 steps with or without a device but without holding on to a rail 3x in 35seconds.

The association of the estimation of having risk for falls with falls having actually occurred is described in Table 2. The estimation of the risk of falls predicted correctly the occurrence of falls in 55% (95%CI 47.5–62.4) of the patients; the positive predictive value of the estimation was 51.1% (95%CI 42.9–59.3) and the negative predictive value was 75% (95%CI 56.7–87.3).

**Figure 2:**
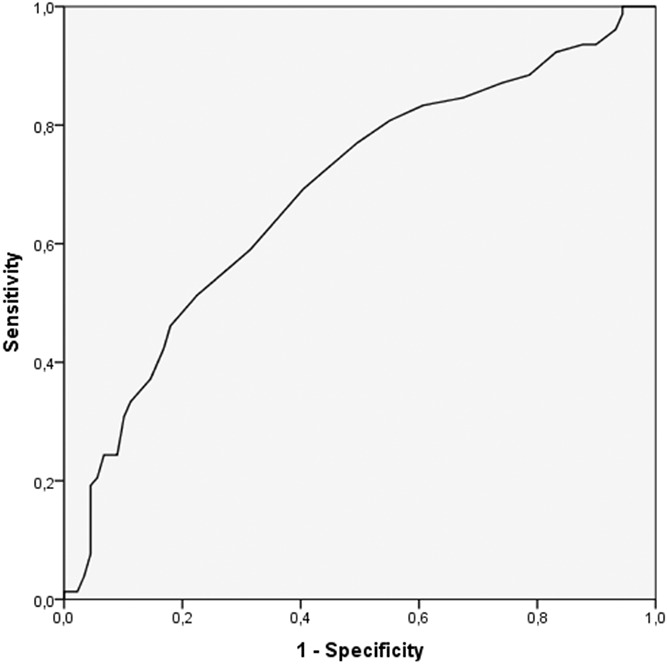
The discrimination ability of the estimation of the actual occurrence of falls by Motor Assessment Scale. AUC 0.69 (95%CI 0.60–0.77). AUC, area under the curve; CI, confidence interval.

No multivariable model for the actual occurrence of falls was identified. The actual occurrence of falls in ambulatory stroke survivors was associated only with Motor Assessment Scale, as a protective factor (for each increase of 1 unit adjusted OR 0.92 [95%CI 0.88–0.9]; *P* < .001). The discrimination ability of the estimation of the actual occurrence of falls by Motor Assessment Scale alone was AUC 0.69 (95%CI 0.60–0.77) (Fig. 2).

## Discussion

In this study, falls occurred in almost half of community-dwelling stroke survivors with an autonomous walk. When the usual instruments are used to identify the risk for falls, they identify a greater proportion of these survivors. The risk of falling is strongly associated with older age, female gender, and a lower Motor Assessment Scale score (in individuals with autonomous gait).

In this study, for most of the sample (79.6%) less than 6 months had passed after stroke and the occurrence of falls was 46.7%. This data is within the values referenced in the literature, indicating that, in the first year after stroke, the percentage of patients who fall may be up to 73%^31^ and that *5*0% of the patients fell at least once in 6 months.^32,33^

The associations found with gender and age are in line both with studies carried out for the general population and those carried out on stroke survivors, which found women and the elderly are at greater risk of falling.^34,35^ It is also known that stroke survivors have not only a greater risk of falling but also a greater number of effective falls.^34^

The risk of falls estimated by the thresholds of Timed Up and Go Test and Berg Balance Scale has a poor performance in discriminating individuals who fell (55%). The search for a model with higher performance, based on the study variables failed; the only variable associated with the actual occurrence of falls was the Motor Assessment Scale (linear), which performs better (69%) than the risk of falls estimated by the thresholds of Timed Up and Go Test and Berg Balance Scale.

Other authors have also found a relationship between functionality and either the risk of falling and/or the occurrence of falls, regardless of the scale used to assess functionality.^33,36–39^

The current study highlights that the estimation of the risk for falls based on the usual tools has low discrimination ability in these Portuguese community-dwelling stroke survivors with autonomous gait. However, it is important to note that in this research only factors related to mobility and balance were considered and risk factors for falls may be related to other key areas.^40,41^ On the other hand, we cannot assume that the patients with greater mobility and balance impairment are the ones who fall the most since, in the presence of severe disabilities, the level of activity is low and the support of others is present during the performance of activities of daily life.

New tools with better performance, covering several dimensions related to the risk for falls in stroke survivors, are necessary to identify the patients with the highest risk, thus allowing the implementation of preventive strategies to be included in the physical therapy intervention. Although several studies have searched for a tool able to correctly identify the risk for falls in various populations^42–45^ and try to define which variables are most related to falls,^39^ the results have not reached consensus.

The results of this observational study, in line with other studies, prove the need for further research in this area. Data collection related to risk factors for the occurrence of falls should be improved (including medication, visuospatial impairments, previous falls, cognitive status, and the existence of architectural barriers) and the description of falls (such as conditions and location) should be included, to allow a holistic approach to this problem.

Patients, who suffered a stroke, especially those over 60 years old, frequently fall. This has serious implications in individual mobility and autonomy, family organization, healthcare resources, and economics, constituting a serious public health problem. Better instruments to assess the risk for falls are needed, allowing effective prevention interventions, both at the individual and environmental levels.

### Strengths and limitations of the study

The main strength of the study is the systematic application of the instruments by experienced professionals, through a standardized protocol. Validated instruments were used, appropriated to the study population and widely recognized as useful in clinical practice and scientific research. The limitations are related to the representativeness of the sample since the recruitment was carried out in individuals referred for physiotherapy, on an outpatient basis, in a tertiary hospital, where the characteristics of the patients may not correspond to the real heterogeneity of all stroke survivors. The small number of patients in the control group, identified as at low estimated risk for falls by Berg Balance Scale or Timed Up and Go Test, has probably affected the ability of the study to identify a significant model for predicting the estimated risk for falls. Even so, none of the exposure variables reached borderline significant association, an indication of the possibility of being significantly associated in a larger sample. Although collected the anatomic location and etiology of the stroke were not consider in the analysis due to data inaccuracy.
